# Inhibition of Hypothalamic MCT4 and MCT1–MCT4 Expressions Affects Food Intake and Alters Orexigenic and Anorexigenic Neuropeptide Expressions

**DOI:** 10.1007/s12035-019-01776-6

**Published:** 2019-10-02

**Authors:** Roberto Elizondo-Vega, Karina Oyarce, Magdiel Salgado, María José Barahona, Antonia Recabal, Patricio Ordenes, Sergio López, Roxana Pincheira, Patricia Luz-Crawford, María Angeles García-Robles

**Affiliations:** 1 Laboratorio de Biología Celular, Departamento de Biología Celular, Facultad de Ciencias Biológicas, Universidad de Concepción, Concepcion, Chile; 2 Facultad de Medicina y Ciencia, Universidad San Sebastián, Concepcion, Chile; 3 Departamento de Bioquímica y Biología Molecular, Facultad de Ciencias Biológicas, Universidad de Concepción, Concepcion, Chile; 4 Laboratorio de Inmunología Celular y Molecular, Centro de Investigación Biomédica, Facultad de Medicina, Universidad de los Andes, Santiago, Chile

**Keywords:** Hypothalamus, Monocarboxylates, Tanycytes, Feeding behavior, POMC neuron

## Abstract

Feeding behavior regulation is a complex process, which depends on the central integration of different signals, such as glucose, leptin, and ghrelin. Recent studies have shown that glial cells known as tanycytes that border the basal third ventricle (3V) detect glucose and then use glucose-derived signaling to inform energy status to arcuate nucleus (ARC) neurons to regulate feeding behavior. Monocarboxylate transporters (MCT) 1 and MCT4 are localized in the cellular processes of tanycytes, which could facilitate monocarboxylate release to orexigenic and anorexigenic neurons. We hypothesize that MCT1 and MCT4 inhibitions could alter the metabolic communication between tanycytes and ARC neurons, affecting feeding behavior. We have previously shown that MCT1 knockdown rats eat more and exhibit altered satiety parameters. Here, we generate MCT4 knockdown rats and MCT1–MCT4 double knockdown rats using adenovirus-mediated transduction of a shRNA into the 3V. Feeding behavior was evaluated in MCT4 and double knockdown animals, and neuropeptide expression in response to intracerebroventricular glucose administration was measured. MCT4 inhibition produced a decrease in food intake, contrary to double knockdown. MCT4 inhibition was accompanied by a decrease in eating rate and mean meal size and an increase in mean meal duration, parameters that are not changed in the double knockdown animals with exception of eating rate. Finally, we observed a loss in glucose regulation of orexigenic neuropeptides and abnormal expression of anorexigenic neuropeptides in response to fasting when these transporters are inhibited. Taken together, these results indicate that MCT1 and MCT4 expressions in tanycytes play a role in feeding behavior regulation.

## Introduction

It has been hypothesized that hypothalamic glucosensing is, at least in part, an indirect process mediated by a metabolic coupling through monocarboxylates between glia and neurons. Tanycytes, which are hypothalamic radial-like glial cells surrounding the walls of the third ventricle (3V), are potentially key players in energy homeostasis. Their position gives them privileged access to the ventricular cerebrospinal fluid (CSF), and their long processes project into the nuclei that control feeding behavior, establishing close contact with anorexigenic and orexigenic neurons of arcuate nucleus (ARC). The ARC is formed by neuronal populations with antagonist functions, which include neurons that inhibit food intake through the release of anorexigenic peptides, such as the α-melanocyte stimulating hormone (α-MSH), a product processed from the pro-opiomelanocortin (POMC), which co-expresses the transcript regulated by amphetamine and cocaine (CART) [1, 2]. This pathway also contains neurons capable of stimulating food intake through the secretion of orexigenic peptides such as neuropeptide Y (NPY) and the peptide associated with agouti (AgRP). In this pathway, also known as the “melanocortin system,” increased NPY and AgRP release and messenger RNA (mRNA) levels are observed with fasting, decreasing after feeding [3]. In contrast, POMC and CART releases are stimulated with feeding [4].

It has been previously shown that tanycytes respond to glucose by increasing intracellular free Ca^2+^ levels, as a result of ATP released through hemichannels [5]. Interestingly, ATP production is dependent on glycolysis and not oxidative phosphorylation. The high glycolytic activity present in tanycytes is further demonstrated by the fact that they release lactate using monocarboxylate transporters (MCTs) [6]. MCT1 and MCT4 located in glial cells have *K*_m_ of 6 and 30 mM respectively, whereas MCT2 is expressed in neurons and has a *K*_m_ of 0.8 mM [7, 8]. MCT2 is involved in the monocarboxylate influx in both anorexigenic and orexigenic neurons, suggesting that monocarboxylates could regulate the activity of these two neuronal types [9]. Interestingly, it has been reported that direct i.c.v. injections of lactate into the hypothalamus suppressed appetite in rats [10].

Recently, we selectively inhibited the expression of MCT1 in tanycytes by injecting adenoviral particles in the 3V, which express a shRNA for MCT1. MCT1 is the main transporter expressed by tanycytes and is located in the short cellular processes of ventral β1-tanycytes, which are in close contact with neurons that express AgRP and NPY. MCT1 knockdown produces a loss in glucose regulation of orexigenic neuropeptides and abnormal anorexigenic neuropeptide expression in response to fasting, accompanied by an increase in food intake and body weight gain [11]. However, tanycytes also express MCT4 to a lesser extent [6] in the long cellular processes of dorsal β1-tanycytes, which are in close contact with neurons that release POMC and CART [6].

Due to the location, as well as kinetics properties, we hypothesized that the inhibition of MCT4 can produce differential alterations in feeding behavior, in comparison with MCT1 [11]. To test this hypothesis, we inhibited the expression of MCT4 (AdshMCT4) in tanycytes using a similar experimental approach. We analyzed changes in feeding behavior during fasting-refeeding cycles, through the determination of meal frequency, inter-meal intervals, meal size, and meal duration. Also, we evaluated the expression of neuropeptides in response to i.c.v. glucose injection after MCT4 or MCT1 and MCT4 inhibitions.

## Results

### MCT4 and MCT1/MCT4 Knockdown in Tanycyte Cultures

**Fig. 1:**
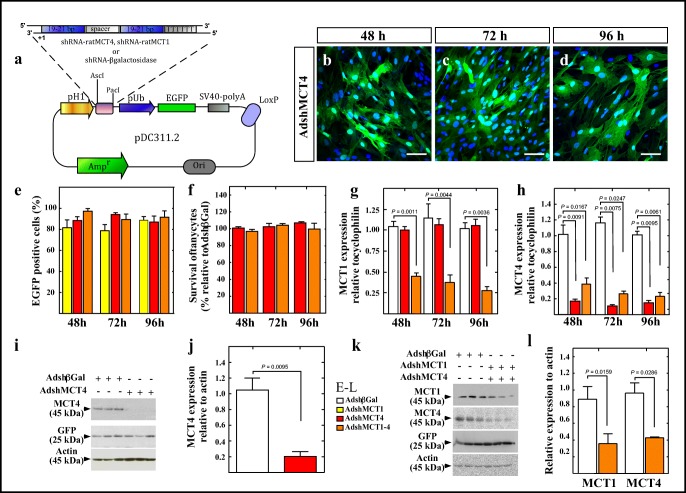
Knockdown of MCT1 and MCT4 in primary cultures of hypothalamic tanycytes. (**a**) The experimental protocol shows the construction of adenoviral shuttle vector. The cassette encoding the H1-promotor, multicloning site (MCS), ubiquitin promoter, EGFP, and SV40 polyA contained in the Fux vector was cloned into the PDC311 adenoviral shuttle expression vector for generating the vector, PDC311.2. The shRNAs were cloned into the MCS. (**b**–**d**) Temporal EGFP expression in tanycyte cultures transduced for 48, 72, and 96 h with AdshMCT4. Nuclei were stained with TOPRO-3 (blue). (**e**) Quantification of EGFP expression normalized to total cells in the tanycyte cultures transduced with AdshMCT1 (yellow bars), AdshMCT4 (red bars), or AdshMCT1 and AdshMCT4 (orange bars) at 48, 72, and 96 h. (**f**) Quantification of survival in tanycytes cultures transduced with AdshMCT4 (red bars) or a mix of AdshMCT1 and AdhMCT4 (orange bars) at 48, 72, and 96 h relative to survival of cells transduced with AdshβGal. (**g**, **h**) Q-RT-PCR analysis of MCT1 (**g**) and MCT4 (**h**) expression in tanycytes infected with AdshβGal (open bars), AdshMCT4 (red bars), or a mix of AdshMCT1 and AdhMCT4 at 48, 72, and 96 h. (**i**, **j**) Western blot (**i**) and semi-quantitative densitometric analysis of MCT4 (**j**) and MCT1–MCT4 (**k**, **l**). Lanes 1–3: total cell extracts treated for 96 h with AdshβGal; lanes 4–6: total cell extracts treated for 96 h with AdshMCT4 (**i**) or a mix of AdshMCT1 and AdshMCT4 (**k**). GFP: transduction control. Actin: loading control. Unpaired *t* test and *p* values were incorporated in the plots. Results represent the mean ± SD of at least four independent experiments. Scale bar 25 μm. MCS multicloning site, pH 1 H1 promoter, pUb ubiquitin promoter, SV40-poly A polyadenylation sequence from Simian virus 40, T4 DNA ligase from bacteriophage

In order to assess the ability of the adenovirus to inhibit MCT4, different viral titers were tested on the HEK 293T cell line, which showed that 5 × 10^7^ IFU/mL was the most effective (nearly 100% transduction) and non-toxic titer (data not shown). The MCT4 adenoviral construct is shown in Fig. 1a. EGFP expression was used to monitor MCT4 adenoviral transduction (Fig. 1b–d), with a high infection rate observed after 72 and 96 h (Fig. 1e). Using a similar protocol, we previously generated AdshMCT1 [11]. In order to use both adenoviruses for in vivo studies, we use half of the concentration for each adenovirus (2.5 × 10^7^ IFU/mL) and evaluated if there was a significant inhibition of both transporters. Cell survival was also measured after adenovirus infection, which showed > 99% of living cells at all times analyzed for MCT4 alone and for both adenoviruses (Fig. 1f), compared to the control condition, transduced with AdshβGal. MCT1 (Fig. 1g) and MCT4 (Fig. 1h) mRNA expressions were quantified after 48, 72, and 96 h post-AdshMCT4 or AdshMCT1–4 transduction by quantitative real-time polymerase chain reaction (Q-RT-PCR). A significant reduction in MCT1 and MCT4 expression levels relative to cyclophilin was detected at all times assayed in the knockdown cultures with respect to controls; no significant differences in MCT4 expression were detected comparing AdshMCT4 knockdown with AdshMCT1–4 knockdowns. The effect of AdshMCT4 alone (I-J) and AdshMCT1–4 (K-L) on MCT1 and MCT4 protein expression was also evaluated in total protein extracts of tanycyte cultures at 96 h post-transduction using EGFP as a transduction control and actin as a loading control. We detected a decrease in both MCT1 and MCT4 band intensity (i.e., protein expression). Quantification revealed that using AdshMCT4 alone resulted in an 81 ± 3.2% MCT4 inhibition when compared with AdshβGal and 66% MCT4 inhibition using both adenoviruses; a similar significant reduction in MCT1 was obtained in this condition. However, because reduction in the protein levels may not necessarily translate to loss in functionality of these transporters, we analyzed lactate uptake and release in primary tanycyte cultures.

### Functional Analysis of Lactate Transport in Tanycytes Cultures Transduced with AdshMCT1 and/or AdshMCT4

**Fig. 2:**
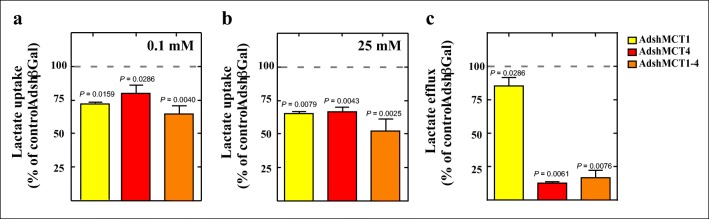
Functional analysis of the MCTs in tanycyte cultures under viral transduction. (**a**, **b**) A total of 0.1 mM l-lactate (**a**) and 25 mM l-lactate (**b**) transport at 4 °C, pH 7.0 at 5 min in tanycytes infected with AdshMCT1 (yellow bar), AdshMCT4 (red bar), or a mix of AdshMCT1 and AdshMCT4 (orange bar), relative to lactate uptake of cells transduced with AdshβGal for 96 h. (**c**) Analysis of lactate efflux over 30 min following incubation with 5 mM glucose in tanycytes infected with AdshMCT1 (yellow bar), AdshMCT4 (red bar), or a mix of AdshMCT1 and AdshMCT4 (orange bar), relative to lactate efflux of cells transduced with AdshβGal for 96 h. Unpaired *t* test and *p* values were incorporated in the plots. Average data represent the mean ± SD of at least four independent experiments

Uptake of 0.1 and 25 mM lactate over 5 min was determined in tanycyte cultures transduced for 96 h with AdshβGal, AdshMCT1, AdshMCT4, and a mix of AdshMCT1 and AdshMCT4. Data was normalized to the uptake of cells transduced with the same titer of control adenovirus. A significant decrease in lactate uptake was observed in MCT1- (Fig. 2a, yellow bar) and MCT4-transduced cells (Fig. 2a, red bar) at 0.1 mM l-lactate. MCT1–MCT4 double inhibitions reduced uptake by 35% (Fig. 2a, orange bar). Using 25 mM l-lactate, where the relative contribution of MCT4 to transport is higher than for MCT1 [8], a significant reduction of uptake was observed after inhibiting MCT1 (Fig. 2b, yellow bar) and MCT4 (Fig. 2b, red bar). However, a higher reduction in lactate uptake was obtained when both transporters were inhibited, reaching a 48% uptake reduction (Fig. 2b, orange bar).

Next, we evaluated if in vitro lactate efflux was inhibited by adenoviruses. MCT1 knockdown reduces lactate release by 14.1 ± 9.4% (Fig. 2c, yellow bar), while MCT4 inhibition decreased lactate release by 88.3 ± 1.8% (Fig. 2c, red bar). This significant reduction in lactate release is maintained when both transporters are inhibited, reaching 83.4 ± 7.5% reduction in release (Fig. 2c, orange bar), compared to AdshβGal control.

### MCT1 and MCT4 In Vivo Inhibition by Adenoviral Injection into the 3V

**Fig. 3:**
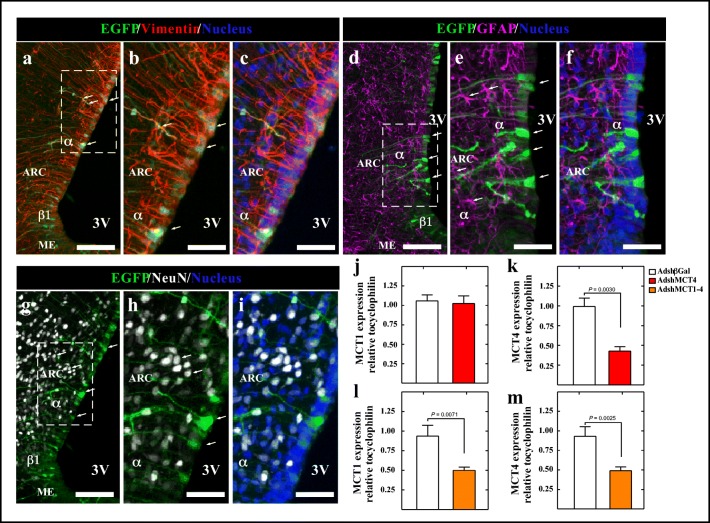
Specificity of adenoviral transduction and MCT mRNA expression in the hypothalamus of rats following i.c.v. injection in the 3V with AdshMCT4 or AdshMCT1 and AdshMCT4. (**a**–**c**) Frontal sections of the hypothalamus (40 μm) in which the immunoreactivity for vimentin (red), EGFP fluorescence (green), and nuclei marker (blue), is shown in rat transduced with AdshβGal at 96 h post-injection (A scale bar 150 μm; B, C scale bar 100 μm). (**d**–**f**) Immunoreactivity for GFAP (magenta), EGFP fluorescence (green), and nuclei marker (blue) is shown in rat transduced with AdshβGal at 96 h post-injection (D scale bar 150 μm; E, F scale bar 100 μm). Co-localization between EGFP and GFAP was not detected. (**g**–**i**) Immunoreactivity for NeuN (white), EGFP fluorescence (green), and nuclei marker (blue) is shown in rat transduced with AdshβGal at 96 h post-injection (**g** scale bar 150 μm; H, I scale bar 100 μm). Co-localization between EGFP and NeuN was not detected. (**j**–**m**) Q-RT-PCR analysis of MCT1 (**j**, **l**) and MCT4 (**k**, **m**) after 96 h of i.c.v. injection of AdshβGal (open bars), AdshMCT4 (red bars), or a mix of AdshMCT1 and AdshMCT4 (orange bars). ARC arcuate nucleus, 3V third ventricle, ME median eminence, β1 β1-tanycytes, β2 β2-tanycytes. Unpaired *t* test and *p* values were incorporated in the plots. Results represent the mean ± SD of at least five independent experiments

Because adenovirus transduction at 96 h in vitro significantly reduced lactate efflux, we used the same condition to evaluate the selectivity and capacity of adenoviruses for reducing the expression of MCTs in vivo. Previously, we have shown that injection of adenoviral particles transduces ependymocytes and tanycytes [11–13]. Frontal sections of the basal hypothalamus of 96-h transduced animals were analyzed by immunofluorescence and spectral confocal microscopy to detect EGFP (green), the tanycyte marker, anti-vimentin (red), the astrocyte marker anti-GFAP (magenta), and the adult neuronal marker NeuN (white) (Fig. 3a–i). EGFP expression was detected in ventricular cells with elongated processes, which due to their location corresponds to α- and β-tanycytes (Fig. 3a, arrows) and with higher magnification exhibiting clear co-localization with vimentin (Fig. 3b, c). Also, these EGFP-positive cells were negative for GFAP at low (Fig. 3d, arrows) and higher magnification (Fig. 3e, f). EGFP expression did not overlap with Neu-N expression (Fig. 3g, arrows), which was most evident at higher magnification (Fig. 3h, i), suggesting the absence of EGFP expression in neurons. Then, we evaluated if MCT1 and MCT4 expressions were affected in vivo after transduction using Q-RT-PCR analysis. MCT4 knockdown rats showed no significant changes for MCT1 mRNA expression (Fig. 3j, red bar) compared with control group (Fig. 3j, open bar) although MCT4 expression was significantly reduced by 56.9 ± 14.9% (Fig. 3k, red bar) compared with control (Fig. 3k, open bar), demonstrating the specificity of shRNA against MCT4. Similarly, the double knockdown showed that the expression of both MCT1 and MCT4 was significantly reduced by 46.8 ± 11.4% (Fig. 3l, orange bar) for MCT1 and 47.4 ± 13.5% for MCT4 (Fig. 3m, orange bar) compared with controls (Fig. 3l, m, open bar).

### Feeding Behavior of Rats Following MCT1 and MCT4 Inhibitions

We have previously reported that 50% inhibition of MCT1 in tanycytes in vivo produces an increase in food intake [11]. Since MCT1 is localized in tanycyte processes that mainly contact orexigenic neurons, while MCT4 is found in tanycyte processes that contact anorexigenic neurons, we evaluated if food intake is altered in knockdown animals for MCT4 or in double knockdown animals in a different way.

**Fig. 4:**
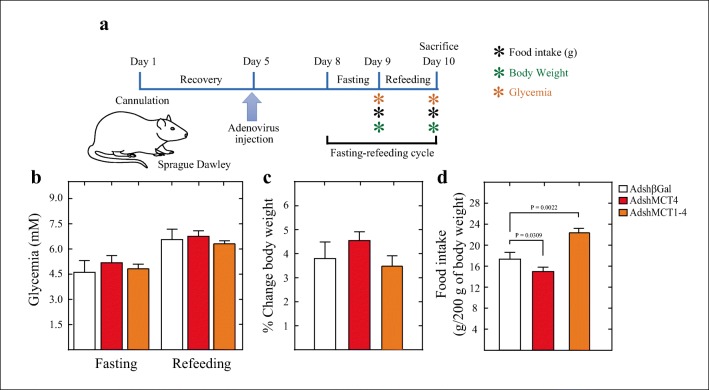
Knockdown of MCT4 decreases food intake while double knockdown of MCT 1 and MCT4 increases food intake. (**a**) Scheme of the experimental protocol. Adult male rats were stereotaxically cannulated into the 3V. After 5 days of recovery, the rats were injected with AdshβGal or a mix of AdshMCT1 and AdshMCT4. At 72 h post-adenoviral injection, the rats were subjected to 24 h of fasting followed by 24 h of refeeding. Parameters, including glycemia, body weight and food intake, were measured. (**b**) Quantification of glycemia after fasting and refeeding in rats transduced with AdshβGal (open bars), AdshMCT4 (red bars), or a mix of AdshMCT1 and AdhsMCT4 (orange bars). (**c**) Percentage of change in body weight at 24 h after refeeding in rats transduced with AdshβGal (open bars), AdshMCT4 (red bars), or a mix of AdshMCT1 and AdhsMCT4 (orange bars). (**d**) Quantification of food intake by rats transduced with AdshβGal (open bars), AdshMCT4 (red bars), or a mix of AdshMCT1 and AdhsMCT4 (orange bars) over 24 h after refeeding and expressed as grams per 200 g of body weight. ns no significance. Unpaired *t* test and *p* values were incorporated in the plots. Average data represent the mean ± SD of at least seven independent experiments

Ten animals per condition were subjected to the experimental protocol shown in Fig. 4a. Briefly, 3 days after adenoviral 3V injection (day 8), rats were exposed to fasting conditions for 24 h (day 9) and then feeding conditions for 24 h (day 10). At the beginning and end of the last 24 h, glycemia, changes in body weight, and food intake were measured (Fig. 4b–d). We did not detect variations in glycemia following MCT4 inhibition or MCT1–MCT4 inhibitions as concentrations remained within normal ranges at all points (Fig. 4b). Similarly, we did not detect changes in body weight (Fig. 4c). However, a significant decrease in food intake was detected in the MCT4 knockdown group (Fig. 4d, red bar) and a significant increase in food intake for the MCT1–MCT4 knockdown groups (Fig. 4d, orange bar), compared with the AdshβGal control group (Fig. 4d, open bar).

**Fig. 5:**
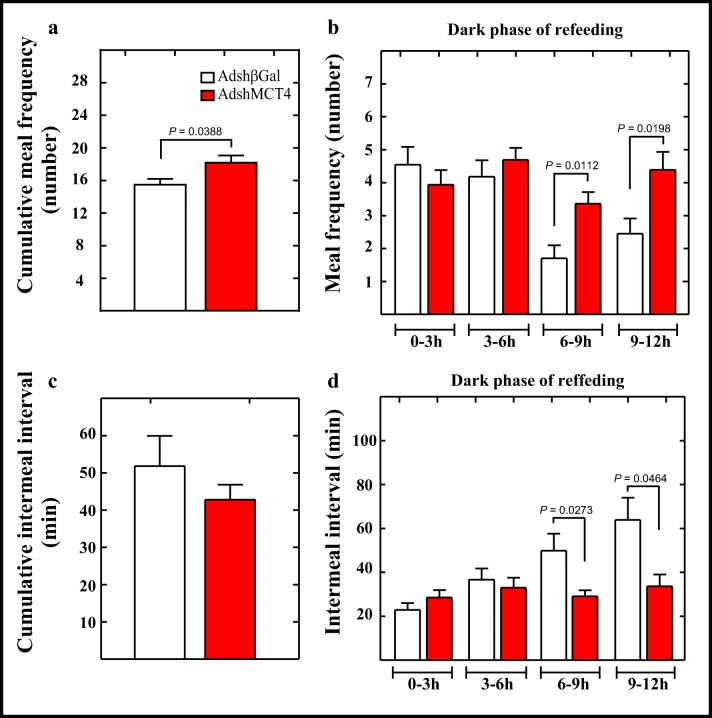
MCT4 inhibition increases meal frequency and reduces satiety. (**a**–**d**) Rats were transduced with AdshβGal (open bars) or AdshMCT4 (red bars) for 96 h, and then animals were subjected to a 24-h period of fasting followed by a 24-h period of refeeding. (**a**) Cumulative meal frequency over 24 h was determined. (**b**) Detailed analysis of meal frequency every 3 h in the dark phase of refeeding. In controls, the meal frequency decreased while remaining relatively constant in AdshMCT4 animals. (**c**) Cumulative inter-meal intervals over 24 h were determined. (**d**) Quantification of inter-meal intervals every 3 h in the dark phase of refeeding. In the controls, the duration of the intervals increased while remaining relatively constant in AdshMCT4 animals, suggesting that MCT4 knockdown disrupted satiety signaling. ANOVA and *p* values were incorporated in the plots. Average data represent the mean ± SD of at least seven independent experiments

We next performed feeding analyses in each individual condition. A general analysis of cumulative meal frequency, defined as the number of times that a feeding event occurs, showed that MCT4 knockdown rats had significantly increased meal frequency than the control group (Fig. 5a, red bar). Cumulative meal frequency was next analyzed in four periods of 3 h, during the dark phase of feeding. During the third (6–9 h) and fourth (9–12 h) periods, a significantly higher meal frequency was observed (Fig. 5b, red bars), which may represent an increase in hunger or a delay in establishing satiety.

Because the shortening of inter-meal intervals suggests an inhibitory effect on satiety, we analyzed the inter-meal intervals in the same periods within the dark phase. A general analysis of inter-meal intervals showed no differences between the MCT4 knockdown rats and the control group (Fig. 5c, red bar). However, a more detailed analysis revealed that during the third and fourth periods, the MCT4 knockdown group had a significantly lower inter-meal interval (Fig. 5d, red bars). Specifically, the inter-meal interval remained constant throughout the dark phase in MCT4 knockdown group; however, it increased in duration during the development of the dark phase in the control group (Fig. 5d, open bars). These data suggest that MCT4 knockdown reduces satiety, but it does not explain the lower food intake in these animals. For this reason, we perform an analysis of the microstructure, which included parameters, such as eating rate, meal duration, and meal size. These parameters were calculated as mean values for the whole cycle, including light and dark phases (Table 1). MCT4 inhibition reduced the eating rate and had a minor effect on mean meal size compared to the control group, which could explain the significant decrease in food intake. However, these animals also showed an increase in mean meal duration (i.e., they stayed longer inside the feeder). Thus, average feeding events were performed at a lower speed for a longer period of time and consuming a smaller amount of food.

**Table 1:** Meal microstructure analysis in MCT4 knockdown and MCT1–MCT4 knockdown rats

Parameter	AdshβGal	SD	AdshMCT4	SD	*p* value	AdshMCT-4	SD	*p* value
Eating rate (mg/min)	130.5	26.1	56.8	17.2	< 0.0001	99.6	25.5	0.0208
Mean meal duration (min)	9.7	2.7	12.8	1.4	0.0020	11.9	1.5	0.0585
Mean meal size (mg)	0.9	0.3	0.6	0.1	0.0091	1.1	0.1	0.0897

Average data represent the mean ± SD of at least seven independent experiments. Unpaired *t* test and *p* values were shown

**Fig. 6:**
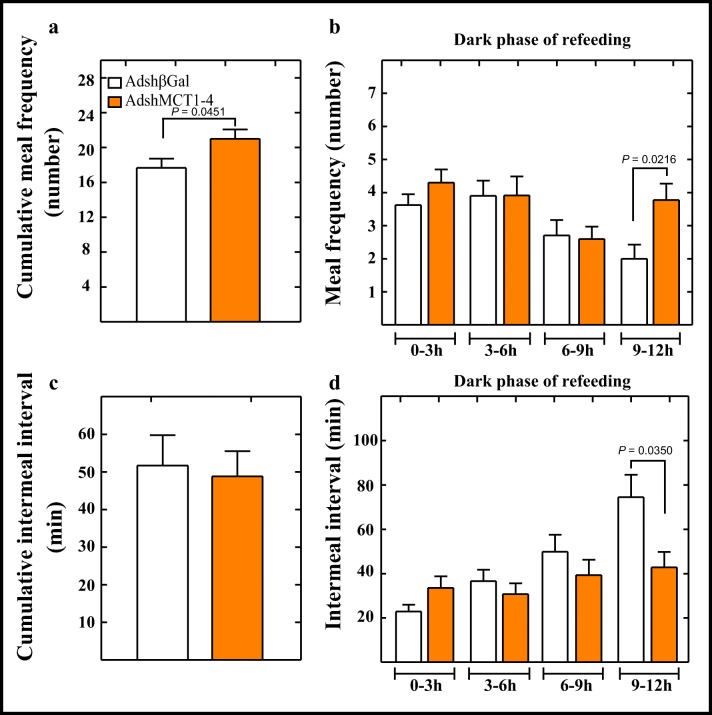
MCT1 and MCT4 knockdowns alter satiety and increase meal frequency. (**a**–**d**) Rats were transduced with AdshβGal (open bars) or AdshMCT1 and AdshMCT4 (orange bars) for 96 h, after which they were subjected to a 24-h period of fasting followed by 24 h of refeeding. (**a**) Cumulative meal frequency over 24 h was determined. (**b**) Detailed analysis of meal frequency every 3 h in the dark phase of refeeding. (**c**) Cumulative inter-meal intervals over 24 h were determined. (**d**) Quantification of inter-meal intervals every 3 h in the dark phase of refeeding. ANOVA and *p* values were incorporated in the plots. Average data represent the mean ± SD of at least seven independent experiments

We next evaluated if the cumulative meal frequency in double knockdown animals was related to an increase in food intake (Fig. 5c, orange bar). The MCT1–MCT4 knockdown groups showed an increase in their cumulative meal frequency (Fig. 6a, orange bar) compared to the control group (Fig. 6a, open bar). Analysis of meal frequency at short periods of times showed that only at the fourth period that double knockdown rats had a significantly higher meal frequency than the control group (Fig. 6b, orange bar), similar to that observed in MCT4 knockdown rats (Fig. 5b, red bar). Analysis of cumulative inter-meal intervals showed no differences between the double knockdown rats and the control group (Fig. 6c, orange bar). However, like the MCT4 knockdown group, the MCT1–MCT4 group showed significantly lower inter-meal interval during the last period (9–12 h) of the dark phase (Fig. 6d, orange bar), remaining constant throughout this time period, compared to control group (Fig. 6d, open bar).

Meal microstructure analysis showed that MCT1–MCT4 knockdown rats had decreased eating rate compared to the control group, but to a lesser degree when compared with the MCT4 knockdown rats (Table 1). No significant differences were detected in the mean meal size with a slight increase in mean meal duration, which was not significant when were compared to control group. Thus, average feeding events were performed at a lower speed for a similar period of time and amount of food compared with control group, but with a higher cumulative meal frequency.

### Neuropeptide Expression in Response to i.c.v. Glucose in Rats Following MCT1 and MCT1–MCT4 Knockdowns

**Fig. 7:**
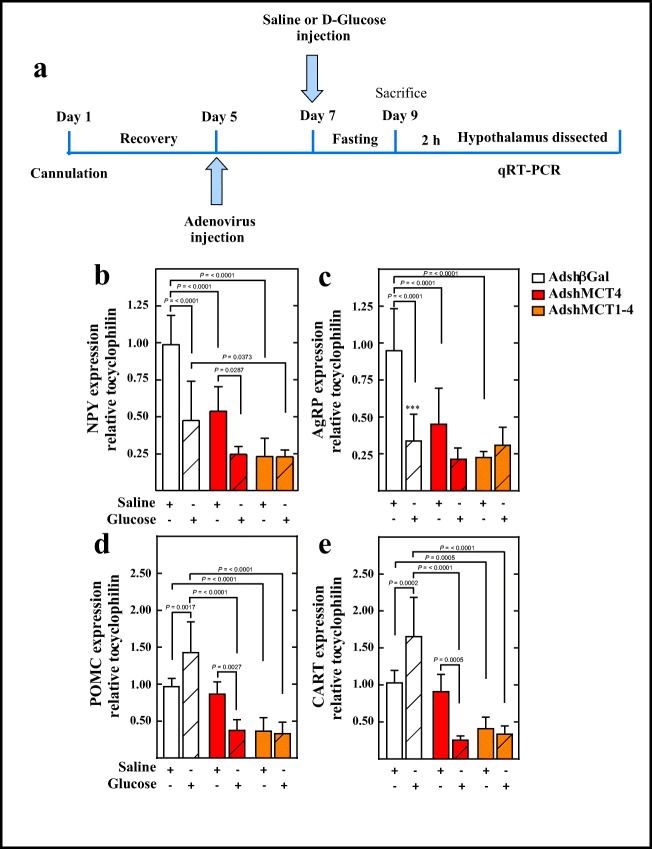
MCT1 and MCT4 inhibitions impair both orexigenic and anorexigenic neuropeptide expressions. (**a**) Scheme of the experimental protocol. Adult male rats were stereotaxically cannulated into the 3V. After 96 h of recovery, the rats were injected with AdshβGal, AdshMCT4, or a mix of AdshMCT1 and AdshMCT4. At 48 h post-injection, the rats were fasted for 48 h and subsequently injected with saline buffer or 50 mM d-glucose (dashed bars). At 2 h post-3V i.c.v. injection, the animals were sacrificed, and the hypothalamus was dissected for RNA extraction and Q-RT-PCR analysis. (**b**–**e**) Q-RT-PCR analysis of NPY (**b**), AgRP (**c**), POMC (**d**), and CART (**e**) neuropeptide expressions in rats transduced with AdshβGal (open bars), AdshMCT4 (red bars), or a mix of AdshMCT1 and AdshMCT4 (orange bars) for 96 h. ANOVA and *p* values were incorporated in the plots. Average data represent the mean ± SD of at least seven independent experiments

It has previously been reported that a 50-mM glucose stimulus directly into the 3V generates a neuronal response mediated by changes in anorexigenic and orexigenic neuropeptide mRNA expressions [11, 12, 14]. Under normal conditions, an increase in 3V glucose concentration decreases the expression of NPY and AgRP neuropeptides; however, in MCT1 knockdown rats, this response does not occur [11]. Therefore, we tested if this glucose response was maintained in MCT4 knockdown rats and in the double knockdown rats, according to the experimental scheme shown in Fig. 7a, which has been used for previous reports [11–14]. Neuropeptide expression was measured by Q-RT-PCR after 2 h of saline (open bars) or d-glucose (dashed bars) i.c.v. injection and normalized to the saline treatment in Adsgβgal knockdown animals (Fig. 7b–e, open bars). After glucose injection, the control AdshβGal group had reduced expression of the orexigenic neuropeptides, reaching a 52% decrease in NPY expression (Fig. 7b, open-dashed bar) and 64.4% decrease in AgRP expression (Fig. 7c, open-dashed bar). In contrast, POMC expression was increased by 42.9% (Fig. 7d, open-dashed bar); CART expression increased by 60.8% (Fig. 7e, open-dashed bar) relative to rats treated with saline, which was similar to that previously reported with this experimental approach [11, 12, 14].

In MCT4 knockdown rats, i.c.v. glucose injection decreased NPY (Fig. 7b, red-dashed bar) and AgRP (Fig. 7c, red-dashed bar) expression by approximately 50% relative to the saline group in a similar way to control conditions (Fig. 7b, c, open bars). However, analysis of MCT1–MCT4 double knockdown in fasting conditions showed a reduced expression of orexigenic neuropeptides, and no differences were observed in response to glucose. These results indicate that (i) glial MCT4 inhibition decreases orexigenic neuropeptide expression in basal conditions compared with AdshβGal rats; (ii) MCT4 knockdown does not alter the response of orexigenic neurons in response to i.c.v. glucose as observed with MCT1 inhibition; and (iii) finally, glial MCT1–MCT4 inhibitions clearly alter the expression of counter-regulatory neuropeptides in both saline and glucose conditions.

In MCT4 knockdown rats, the expression of anorexigenic neuropeptides in response to glucose differs from that observed in the control group; POMC decreased by 61.6% (Fig. 7d, red-dashed bar), and CART decreased by 66.7% (Fig. 7e, red-dashed bar) in MCT4 knockdown rats. The MCT1–MCT4 knockdown groups again showed a loss in the regulatory expression of anorexigenic neuropeptides with reduced POMC (Fig. 7d, orange bar) and CART (Fig. 7e, orange bar) expression without changes in response to glucose. These results suggest that responses of anorexigenic neurons to i.c.v. glucose depend on monocarboxylates released by tanycytes through MCT4, while double knockdown animals showed a complete alteration of neuropeptide expression in response to i.c.v. glucose administration and in fasting conditions (Fig. 7d, e, orange-dashed bars).

## Discussion

We have previously demonstrated that MCT4 is located in the long cellular processes of dorsal β1-tanycytes, which are in close contact with anorexigenic neurons, while MCT1-positive processes preferentially contact orexigenic neurons [6]. Due their location, we hypothesized that MCT4 inhibition could alter feeding behavior, in comparison with MCT1 [11]. Our results showed that MCT4 knockdown in rats decreased their food intake, which is opposite to that reported in MCT1 knockdown rats [11]. MCT4 inhibition in tanycytes also decreased the expression of NPY and AGRP under fasting conditions, which could explain the reduction in food intake; however, their expression did not change in response to glucose. However, MCT4 inhibition shortened meal intervals, which is associated with decreased satiety and increased number of feeding events, all of which results in increased hunger. These results suggest that the animals are less satiated after each meal event, resulting in increased meal frequency. The lower food intake can be explained by analyzing the eating behavior microstructure, in which the animals eat at half the eating rate of controls.

Feeding behavior is a complex process with several interconnected regulating mechanisms. Thus, the alterations produced by MCT4 knockdown cannot be explained solely by impaired lactate flux between tanycytes and anorexigenic neurons. Recent studies show that other monocarboxylates might also affect anorexigenic or orexigenic neurons though their binding to ketone body receptors [15, 16]. In addition to their role as metabolic and energy substrates, monocarboxylates can act as signaling molecules through specific G protein-coupled hydroxycarboxylic acid receptors (HCARs) [17]. In rodents, two inhibitory G-coupled receptors subtypes have been described, HCA1R which is activated by physiologic concentrations of lactate, and HCA2R, which responds selectively to β-hydroxybutyrate. Recently, the expression of these receptors has been shown in neurons of the adult brain [17], suggesting that they could influence neuronal activity [17, 18]. On the other hand, immunolocalization studies have shown high levels of MCT2 in the membrane of ARC neurons and are not detected in tanycytes [9]. MCT2 expression is upregulated by an increase in extracellular monocarboxylate concentrations [19]; therefore, it is possible that MCT1 and MCT4 glial inhibitions could lead to a reduction in MCT2 neuronal expression [11]. In this aspect, further studies will be necessary to evaluate the neuronal function of MCT2 on food intake.

We previously reported that MCT1 inhibition in tanycytes reduced hunger sensations at the beginning of the dark phase, which could be associated with a minor food anticipation phenomenon, a process known to be regulated by ketone bodies [20]. This loss of food intake anticipation does not occur in MCT4 knockdown animals, which could be explained by the fact that ketone bodies can be transported by MCT1 but not MCT4 [8, 21], suggesting that MCT4 in tanycytes does not participate in food intake anticipation.

The opposite results observed between MCT1 and MCT4 knockdown animals suggested that if both transporters were inhibited, compensatory responses may occur when evaluating feeding behavior. However, both MCT1 inhibition [11] and MCT1–MCT4 double inhibition led to an increase in food intake, masking the effects of MCT4. This can be attributed to MCT1 being the main transporter expressed by tanycytes [6] and to the fact that MCT4-inhibitted animals also showed satiety inhibition.

A more detailed analysis of feeding behavior in double knockdown animals revealed that they have increased food intake, similar to MCT1-inhibited animals, but exhibit increased cumulative meal frequency similar to MCT4 inhibition; they also had reduced eating rate, although it was less significant than MCT4-inhibited animals. We believe that food intake velocity in double knockdown animals is the result of compensatory effects between MCT1 and MCT4 inhibition, as we previously reported that MCT1 inhibition slightly increased food intake rate [11]. Thus, the inhibition of both transporters generates combined feeding behavior responses as well as severely altered the expression of neuropeptides, resulting in loss of satiety establishment.

The increase of food intake observed in double knockdown animals agrees with previous studies where proteins involved in glucosensing are inhibited in vivo, specifically in tanycytes. GLUT2 and GK inhibitions in tanycytes also increase food intake, which also altered the expression of orexigenic and anorexigenic neuropeptides under increased glycorrhachia [12, 13]. Based on these recent reports, we propose that an alteration in glycolytic metabolism, either by reducing the incorporation, phosphorylation, or metabolization of glucose to lactate in tanycytes, participates in modulating food intake, due at least in part, to changes in the function of neuroendocrine neurons from the ARC.

Altogether, these data support a metabolic signaling between tanycytes and neurons that participate in the regulation of feeding behavior under physiological conditions induced by glucose and mediated by lactate. It would be interesting to know if this interaction is altered in pathologies that involve changes in cell metabolism, such as diabetes and obesity.

## Materials and Methods

### Ethics Statement

All studies performed using animals were in strict accordance with the Guidance on the Operation of the Animals (Scientific Procedures) Act 1986 and approved by Animal Ethics Committee of the Chile’s National Commission for Scientific and Technological Research (CONICYT, protocol for project no. 1180871) and the appropriate Ethics and Animal Care and Use Committee of the Universidad de Concepción, Chile (permit number 2010101A). Male adult Sprague-Dawley rats weighing 250–300 g were used in all experiments. Animals were housed in a separate animal room with constant temperature (21 ± 2 °C) and a controlled 12-h light/ 12-h dark cycle; lights were turned on every day at 7:00 a.m. Animals were fed ad libitum with a standard rodent diet (Lab Diet, 5P00 Prolab RMH 3000, Purina Mills, St. Louis, MO) and had free access to tap water.

### Preparations of Adenoviral shRNA-MCT1 Vectors

DNA sequences were designed with siDESIGN Center software (Dharmacon RNAi Technologies, Lafayette, CO, USA) in order to target rat MCT1 (GenBank: D63834.1) and MCT4 (Gene ID: 295356) transcriptional expression. Using Blast, those sequences targeting the expression of other rat genes were discarded from the selection. Primer sets were validated using dissociation curves and used previously in Cortes-Campos et al. [6], Cortes-Campos et al. [9], and Elizondo-Vega et al. [11]. The following sets of oligonucleotides were used: MCT1 sense 5′-CGC GCC GCA GCT TCT TTC TGT AAC ATT CAA GAG ATG TTA CAG AAA GAA GCT GCT TTT TTT TAA T-3′ and MCT1 antisense 5′-TAA AAA AAA GCA GCT TCT TTC TGT AAC ATC TCT TGA ATG TTA CAG AAA GAA GCT GCG G-3′, MCT4 sense 5′-CGC GCC GGG ATT GGC TAC AGC GAC ATT CAA GAG ATG TCG CTG TAG CCA ATC CCT TTT TTT TAA T-3′ and MCT4 antisense 5′-TAA AAA AAA GGG ATT GGC TAC AGC GAC ATC TCT TGA ATG TCG CTG TAG CCA ATC CCG G-3′. A ring sequence of nine base pairs (TTC AAG AGA) was placed between the sense and antisense strands. Control siRNA oligonucleotides were designed and selected to target β-galactosidase from *E. coli:* sense 5′-CGC GCC AAG GCC AGA CGC GAA TTA TTT CAA GAG AAT AAT TCG CGT CTG GCC TTT TTT TTT TAA T-3′ and antisense 5′-TAA AAA AAA AAG GCC AGA CGC GAA TTA TTC TCT TGA AAT AAT TCG CGT CTG GCC TTG G-3′. Cloning of the expression cassette into the adenoviral shuttle vector was performed as previously reported [11, 12], inserting the shRNA coding sequence into the multicloning site through the AscI and PacI sites. The adenoviral expression system was produced in HEK293A cells by the cotransfection of pBHGlox(Δ)E1,3Cre (Admax system, Microbix biosystems Inc., Ontario, Canada) adenoviral genomic DNA and either pDC311-H1-shMCT1-Ub-EGFP, pDC311-H1-shMCT4-Ub-EGFP, or the pDC311-H1-shβGal-Ub-EGFP expression vectors. The resulting adenoviral expression vectors were titered by EGFP expression using the Adeno-XTM Rapid Titer Kit Protocol (Clontech Laboratories, Inc., CA, USA). After amplification, adenoviral particles were purified using the VirakitAdenoMini-4 kit (Virapur, CA, USA), aliquoted, and stored at − 80 °C.

### Primary Culture of Tanycytes

Cultures of hypothalamic tanycytes were isolated following the method described previously [5, 6, 22, 23]. One-day postnatal rats were rapidly decapitated, the brains were removed, and the region close to the ventricular region was dissected in the cold. Samples were incubated with 0.25% trypsin-0.2% EDTA (w/v) for 20 min at 37 °C, before transfer to MEM media ((Invitrogen, Carlsbad, CA, USA) supplemented with 10% (v/v) fetal bovine serum (FBS) (Thermo Fisher Scientific Inc., Waltham, MA, USA), 2 mM l-glutamine, 100 U/mL penicillin, 100 μg/mL streptomycin (Thermo Fisher Scientific, Auckland, NZ), and 2 mg/mL DNase I (Sigma-Aldrich, St. Louis, MO, USA). For subsequent experimental procedures, tanycytes were washed twice in 0.1 M phosphate buffer solution (PBS), pH 7.4, and treated with 0.25% trypsin-0.2% EDTA for 3 min at 37 °C. Dishes with the highest density of confluent epithelial cells were expanded for subsequent adenoviral transduction to measure cell survival, transduction efficiency, and protein expression.

### Adenoviral Transduction In Vitro

To measure cell survival and transduction efficiency, cells were grown on poly-l-lysine-coated glass cover slides in 24-well plates in MEM medium supplemented with 10% (v/v) FBS. Cells were infected with Ad-MCT1-shRNA, Ad-MCT4-shRNA, Ad-βGal-shRNA (control), or a mix of Ad-MCT1-shRNA and Ad-MCT4-shRNA at 5 × 10^7^ infectious units per mL (IFU/mL). Virus-containing medium was replaced 24 h later with MEM medium containing 10% (v/v) FBS and incubated for a total of 48, 72, and 96 h. Survival was measured by the Trypan Blue 0.4% (Thermofisher) viability assay. After fixation with 4% paraformaldehyde (PFA) and visualization using a confocal microscopy LSM 700 (Zeiss, Oberkochen, Germany), the percentage of transduction was calculated as the number of EGFP-positive cells over the total of cells using TOPRO-3 (1:1000, Invitrogen) nuclear staining.

### Cannula Implantation

Cannulas were stereotaxically implanted into the 3V with the following protocol. Rats were anesthetized with an intraperitoneal injection of ketamine-xylazine (90–10 mg/kg), and the fur at the top of the head was removed to expose the area to be incised. A hole was drilled in the skull, and a guide cannula (28 gauge stainless steel; Plastics One, Roanoke, VA) was lowered using the following stereotaxic coordinates: anterior-posterior from bregma − 3.14 mm, medial-lateral from midsaggital sinus 0.0, and dorsal-ventral from the top of the skull 9.2 mm. The guide cannula was secured to the skull using 3/32 mm mounting screws and dental acrylic. A removable dummy cannula (28 gauge stainless steel; Plastics One, Roanoke, VA) was placed into the cannula guide, sealing the opening in the guide cannula throughout the experiments except when it was removed for the injections. Rats were housed individually following surgery and allowed to recover for 5 days before adenovirus administration and starting the experimental procedures.

### I.c.v. Injections of AdshMCT4, AdshβGal, or both AdshMCT1 and AdshMCT4 Adenoviruses

Rats were anesthetized with isoflurane and then injected into the 3V with 30 μL of 2 × 10^9^ IFU/mL. For mRNA expression analysis, rats were injected with adenovirus as described in Fig. 7a. Subsequently, the rats were anesthetized with isoflurane and injected with 10 μL of saline buffer (128 mM NaCl, 3 mM KCl, 1.3 mM CaCl_2_, 1.0 mM MgCl_2_, 1.3 mM NaH_2_PO_4_, 21 mM Na_2_HPO_4_, pH 7.4 and 320 mOsm) or 10 μL of 50 mM d-glucose diluted in the same buffer (320 mOsm, pH 7.4). Hypothalamic samples were collected after 2 h post-glucose or saline injection for the mRNA expression analysis. For protein analysis, hypothalamic samples were collected at 96 h post-adenoviral injection. At 72 h post-adenoviral injection, rats were subjected to one cycle of a 24-h fasting period followed by 24-h refeeding period for the feeding behavior analysis.

### Measurement of hypothalamic mRNA

A-Q-RT-PCR analysis was used to measure the expression of the hypothalamic cyclophilin, MCT1, MCT4, NPY, AgRP, POMC, and CART. First, the brain of each rat was removed, and hypothalamic areas (Bregma − 1.74/− 4.56) close to the 3V were isolated and further dissected. Total RNA from hypothalamic samples was isolated using TRIZOL (Life Technologies, Carlsbad, CA) and treated with DNase I (Fermentas International, Burlington, Ontario, Canada). RT-PCR was performed according to the manufacturer’s protocol (Fermentas International) using 2 μg of RNA and 20 μL reaction volume containing 10 mM Tris-HCl (pH 8.3), 50 mM KCl, 5 mM MgCl_2_, 20 U RNase inhibitor, 1 mM dNTPs, 2.5 μM of oligo d(T) primers, and 50 units of MuLV reverse transcriptase (New England Biolabs, Ipswich, MA, USA) for 60 min at 42 °C followed by 10 min at 70 °C. Parallel reactions were performed in the absence of reverse transcriptase to control for the presence of genomic DNA. Q-RT-PCR reactions were prepared with 1x Brilliant II SYBR Green qPCR Master Mix kit (Agilent Technologies, Santa Clara, CA, USA) in a final volume of 12.5 μL containing 1 μL cDNA and the following sets of primers (500 nM each): cyclophilin, sense 5′-ATA ATG GCA CTG GTG GCA AGT C-3′ and antisense 5′-ATT CCT GGA CCC AAA ACG CTC C-3′; MCT1, sense 5′-TGG AAT GTT GTC CTG TCC TCC TGG-3′ and antisense 5′-TCC TCC GCT TTC TGT TCT TTG GC-3′; MCT4, sense 5′-TTC TCC AGT GCC ATT GGT CTC GTG-3′ and antisense 5′-CCC GCC AGG ATG AAC ACA TAC TTG-3′; NPY, sense 5′-TGT TTG GGC ATT CTG GCT GAG G-3′ and antisense 5′- CTG GGG GCA TTT TCT GTG CTT TC-3′; AGRP, sense 5′-GCA GAC CGA GCA GAA GAT GTT C-3′ and antisense 5′- GTA GCA CGT CTT GAA GAA GCG G-3′; POMC, sense 5′-CTC CTG CTT CAG ACC TCC ATA GAC-3′ and antisense 5′-AAG GGC TGT TCA TCT CCG TTG-3′; and CART, sense 5′-TCT GGG AAG AAG AGG GAC TTT CGC-3′ and antisense 5′-TCC ATT TGT GTT GCT TTG GGG TG-3′. All reactions were performed with an initial denaturation of 5 min at 95 °C, followed by 40 cycles of 30 s at 95 °C, annealing for 30 s at 55 °C, and extension for 1 min at 72 °C. The relative changes in gene expression were calculated by the relative quantification method (2^−ΔΔCt^) and normalized according to the expression in control conditions.

### Immunoblotting

Total protein extracts were obtained from rat hypothalamic samples and primary cultures of tanycytes. Samples were homogenized in protease inhibitor cocktail (ROCHE) and sonicated three times on ice at 300 W. Proteins were resolved by SDS-PAGE (50 μg/lane) in a 5–15% (w/v) polyacrylamide gel, transferred to PVDF membranes (0.45 μm pore, Amersham Pharmacia Biotech., Piscataway, NJ, USA), and probed overnight at 4 °C with the following antibodies: chicken anti-MCT1 (1:1000, MERCK, Darmstadt, Germany), chicken anti-MCT4 (1:1000, MERCK, Darmstadt, Germany), and mouse anti-β-actin (1:10,000; Santa Cruz). After extensive washing, the PVDF membranes were incubated for 2 h at 4 °C with peroxidase-labeled rabbit anti-chicken or anti-goat (1:1000; Jackson ImmunoResearch Laboratories, Inc., PA, USA) secondary antibodies. The reaction was carried out using the enhanced chemiluminescence (ECL) Western blot analysis system (Amersham Biosciences). Images shown are representative of at least three samples originated from at least three separate experiments. β-Actin expression levels were used as a loading control for densitometric analysis.

### Immunocytochemistry

In order to analyze the specificity of the adenovirus in vivo, the animals were injected with the adenovirus, and the brains were collected at 48 and 96 h. The rat brains were fixed in 4% PFA by immersion for 48 h. Free-floating frontal hypothalamic slices of 40 μm thickness were obtained by a cryostat and subsequently processed. Tissues were stained with chicken anti-vimentin (1:200; Millipore, Billerica, MA, USA), mouse anti-GFAP (1:200; Millipore), and rabbit anti-NeuN (1:5000; Abcam, Cambridge, MA, USA) antibody diluted in Tris-HCl buffer (pH 7.8) containing 1% bovine serum albumin. After extensive rinsing, the sections were incubated for 2 h at room temperature with Cy2- or Cy3-labeled secondary antibodies (1:200; Jackson ImmunoResearch Laboratories). TOPRO-3 (1:1000; Invitrogen) was used as nuclei staining. The slides were visualized and captured using confocal laser microscopy LSM 700 (Zeiss).

### Lactate Uptake and Efflux Analysis

For lactate uptake and efflux assays, we following the method described previously [6]. Briefly, tanycyte primary cultures were washed and placed in incubation buffer (15 mM HEPES [pH 7.0], 135 mM NaCl, 5 mM KCl, 1.8 mM CaCl_2_, 0.8 mM MgCl_2_, 320 mOsm) for 10 min at room temperature. Uptake assays were performed in 0.2 mL of incubation buffer at 4 °C with two different l-lactate (Sigma-Aldrich) concentrations (0.1 and 25 mM) and 1–4 μCi of L-[14C(U)] lactic acid sodium salt (> 100 mCi [3.70GBq]/mmol; PerkinElmer-NEN, Boston, MA, USA). Uptake was stopped by washing the cells with ice-cold stop buffer (incubation buffer plus 1 mM HgCl_2_). Cells were lysed in 0.4 mL of lysis buffer (10 mM Tris-HCl [pH 8.0], 0.2% SDS), and the incorporated radioactivity was quantified by liquid scintillation counting.

For lactate release assays, cells were washed with 0.1 M PBS and incubated for several times in efflux buffer (44 mM sucrose, 10 mM HEPES [pH 7.4], 135 mM NaCl, 5 mM KCl, 0.15 mM Na_2_HPO_4_, 0.2 mM KH_2_PO_4_, and 5 mM glucose). Supernatant was removed and assayed for lactate determination using a high-performance liquid chromatography (HPLC) system from Merck Hitachi (Merck, Darmstadt, Germany), consisting in an L-6200 pump and a Hitachi L-4200 UV-VIS (225 nm) detector. Samples were separated by chromatography on an Aminex HPX-87H column (Bio-Rad Laboratories, Hercules, CA, USA) of 300 × 7.8 mm. The mobile phase consisted of an isocratic solution of 20 mM H_2_SO_4_. The l-lactate peak was identified by comparison of its retention time with that of a reference standard, and its concentration was quantified using the area under the peak (Merck Hitachi D-2500 chromato-integrator).

### Measurement of Blood Glucose

Blood samples for glucose measurements were taken by needle puncture from the tail vein after 24 h in a fasting condition to ensure a hypoglycemic state. Blood glucose measurements performed on whole blood were made with an Accu-Chek Go (Roche) glucometer.

### Measurement of Food Intake

Rats were handled for 1 week each day to become accustomed to the researchers and experimental procedures. This included removal of the rats from the cage to measure food intake and body weight. Food intake was quantified by providing rats a defined mass of chow and weighing the food not consumed food after a defined time interval. Food intake was expressed as grams consumed per 200 g of body weight (g/200 g body weight). Every interaction with the feeder was recorded by a computerized data acquisition system (VitalView, Respironics, Inc., Murraysville, PA, USA). A meal was defined as a bout at the feeder that was larger than 5 s, and these events were separated from other feeding bouts by more than 10 min of inter-meal interval [15, 24]. When bouts of feeding were longer than 30 min, they were considered two meal events. The meal pattern parameters were calculated as follows: inter-meal interval (min), meal frequency (number), cumulative meal frequency (number), mean meal size (mg/meal), mean meal duration (min/meal), and eating rate (mg/min). The inter-meal interval was calculated as the period between the end of one meal and the initiation of the subsequent one. The cumulative meal frequency was defined as the total meals in 24 h. The mean meal size was determined as the total food intake (mg) divided by frequency. The mean meal duration was calculated by dividing the total meal duration (min) by meal frequency, and the eating rate was estimated by dividing total food intake (mg) by total meal duration (min).

### Statistical Analyses

For statistical analysis, each treatment was compared to its respective control. Significant differences were determined using the Student’s *t* test and Mann-Whitney post-hoc *U* test or one-way ANOVA with multiple comparison test. Differences were considered significant when *p* < 0.05 using GraphPad Prism 5.0 Software (GraphPad Software Inc., San Diego, CA, USA). Results were expressed as mean ± standard deviation (SD), and *n* refers to the number of animals used.
